# Chlorin e6 Prevents ADP-Induced Platelet Aggregation by Decreasing PI3K-Akt Phosphorylation and Promoting cAMP Production

**DOI:** 10.1155/2013/569160

**Published:** 2013-08-13

**Authors:** Ji Young Park, Hyun Dong Ji, Bo Ra Jeon, Eun Ju Im, Young Min Son, Joo Young Lee, Dong-Ha Lee, Young-Chul Lee, Eujin Hyun, Qi Jia, Mei Hong, Hwa-Jin Park, Man Hee Rhee

**Affiliations:** ^1^Laboratory of Veterinary Physiology and Cell Signaling, College of Veterinary Medicine, Kyungpook National University, Daegu 702-701, Republic of Korea; ^2^Department of Biomedical Laboratory Science, College of Biomedical Science and Engineering and Regional Research Center, Inje University, Gimhae 621-749, Republic of Korea; ^3^Unigen Inc., Cheonan, Chungnam 330-863, Republic of Korea

## Abstract

A number of reagents that prevent thrombosis have been developed but were found to have serious side effects. Therefore, we sought to identify complementary and alternative medicinal materials that are safe and have long-term efficacy. In the present studies, we have assessed the ability of chlorine e6 (CE6) to inhibit ADP-induced aggregation of rat platelets and elucidated the underlying mechanism. CE6 inhibited platelet aggregation induced by 10 *µ*M ADP in a concentration-dependent manner and decreased intracellular calcium mobilization and granule secretion (i.e., ATP and serotonin release). Western blotting revealed that CE6 strongly inhibited the phosphorylations of PI3K, Akt, c-Jun N-terminal kinase (JNK), and different mitogen-activated protein kinases (MAPKs) including extracellular signal-regulated kinase 1/2 (ERK1/2) as well as p38-MAPK. Our study also demonstrated that CE6 significantly elevated intracellular cAMP levels and decreased thromboxane A_2_ formation in a concentration-dependent manner. Furthermore, we determined that CE6 initiated the activation of PKA, an effector of cAMP. Taken together, our findings indicate that CE6 may inhibit ADP-induced platelet activation by elevating cAMP levels and suppressing PI3K/Akt activity. Finally, these results suggest that CE6 could be developed as therapeutic agent that helps prevent thrombosis and ischemia.

## 1. Introduction

Vascular structure integrity is essential for the maintenance of cardiovascular function. Exposure to an injured vascular wall triggers the activation of platelets to promote the recovery of blood vessel integrity. However, aberrant platelet activation may result in thrombosis that could lead to the development of serious vascular diseases such as cerebral stroke and myocardial infarction.

Platelet agonists (e.g., ADP, thrombin, thromboxane A_2_ (TXA_2_), and epinephrine) activate platelets via G protein-coupled receptor (GPCR) pathways [1, 2], and the activations of distinct G proteins are integrated by interacting downstream molecules [3, 4]. Thus, once a specific GPCR is activated by an agonist, other GPCRs are eventually activated by recruited GPCR ligands and form a positive feedback loop which greatly amplifies activation signals [1, 5]. ADP and TXA_2_, two factors secreted during platelet activation, serve as both mediators and agonists. On the other hand, cyclic adenosine monophosphate (cAMP) and nitric oxide (NO), an endogenous gaseous molecule, have the opposite effects on platelet activation [2, 6, 7]. Adenylyl cyclase (AC) catalyzes the conversion of 5′-ATP into cAMP whereas phosphodiesterase (PDE) hydrolyzes cAMP. Intracellular cAMP levels are thus tightly regulated by AC and PDE [8].

A number of events involved in platelet activation are largely dependent on intracellular calcium levels ([Ca^2+^]_*i*_) [9], and elevation of [Ca^2+^]_*i*_ is considered to be a prerequisite for platelet aggregation. The final common event of platelet aggregation is the binding of fibrinogen to integrin *α*_IIb_ *β*_3_. The activation of integrin *α*_IIb_ *β*_3_ mediates outside-in signaling that stimulates tyrosine phosphorylations to complete the process of platelet aggregation [10]. As such, platelet agonists, such as ADP and collagen, increase the affinity of *α*_IIb_ *β*_3_ for fibrinogen that forms crosslinks between platelets [11].

Platelet activation triggers hemostasis, and the aberrant activation of platelets leads to thrombotic complications [12, 13]. Thrombosis is the formation of blood clots that can obstruct blood flow in a vessel. When thrombi form in a vessel, tissues can become hypoxic. This might lead to the development of serious thrombotic diseases including cerebral stroke and myocardial infarction [14, 15].

Over the last couple of decades, a number of antiplatelets drugs have been developed in response to the growing concern about cardiovascular diseases. However, these drugs were found to have undesirable side effects resulting from the direct inhibition of clotting factors. In our effort to find complementary and alternative compounds, we discovered CE6, a natural product that is made by chlorella species [16]. These algae have been used as traditional medicine in India [17] and Turkey [18] for treating inflammation-related diseases. Previous studies have also reported that CE6 has anticancer [19] and antiviral [20] effects. McMahon et al. [21] have examined the effects of CE6 on the changes of blood flow by measuring the diameters of cremaster arterioles *ex vivo*. Samal' et al. also described the antithrombotic effects of CE6 [22], but the underlying mechanism affecting the antiplatelet activities of this compound has not been elucidated. Therefore, we performed the current study to investigate the inhibitory effect of CE6 on ADP-induced platelet aggregation and the associated signaling mechanism governing this activity.

## 2. Materials and Methods

### 2.1. Materials

**Figure 1:**
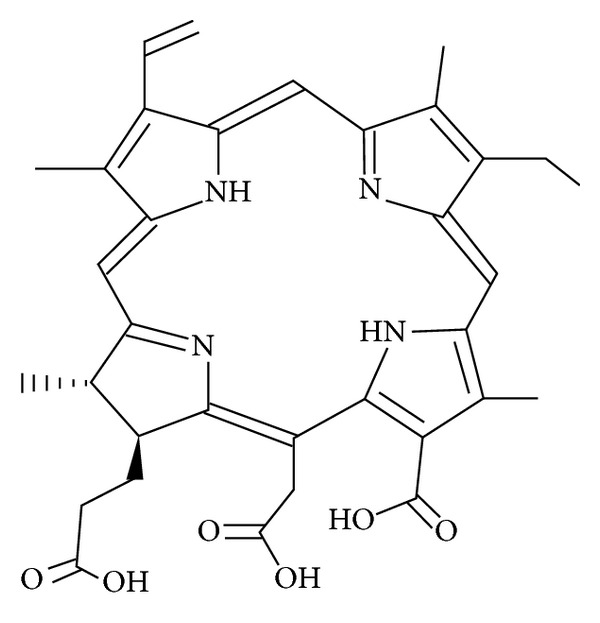
The chemical structure of CE6.

CE6 was obtained from Frontier Scientific, Inc. (Figure 1, Logan, UT, USA). Purity of the CE6 sample was more than 95%. A 50 mM stock solution of CE6 was prepared in DMSO and stored at −20°C in the dark. The CE6 was diluted to the appropriate concentration immediately before all experiments were performed. 

Fura-2/AM was obtained from Sigma Chemical Co. (St. Louis, MO, USA). ADP was procured from Chrono-log (Havertown, PA, USA). Antibodies against phospho-p44/42, p44/42, phospho-p38, p38, phospho-SAPK/JNK, phospho-PI3K (p85), phospho-Akt, and *β*-actin were acquired from Cell Signaling (Beverly, MA, USA). Antibody against the catalytic subunit of PKA (PKA*α*/*β*/*γ* cat) was from Santa Cruz (Delaware Avenue, CA, USA). ATP assay kits were purchased from Biomedical Research Service Center (Buffalo, NY, USA). A TXB_2_ enzyme immunoassay (EIA) kit was purchased from Enzo Life Sciences (Plymouth Meeting, PA, USA). A cAMP EIA kit was obtained from Cayman Chemical (Ann Arbor, MI, USA). Fibrinogen Alexa Fluor 488 conjugate was obtained from Molecular Probes (Eugene, OR, USA). All chemicals were of reagent grade.

### 2.2. Rat Platelet Preparation

Rat platelets were isolated and prepared as previously described [23]. Male Sprague Dawley rats 60 d old and weighing from 240 to 250 g were obtained from Orient Co. (Seoul, Republic of Korea). The animals were maintained in a standard laboratory animal facility with free access to food and water. Whole blood from the rats was collected using a 23 G needle inserted into the abdominal aorta, and then transferred to a 15 mL test tube containing 1 mL of an acid/citrate/dextrose (ACD) solution (85 mM trisodium citrate, 83 mM dextrose, and 21 mM citric acid) as an anticoagulant. The blood was centrifuged at 170 ×g for 7 min to obtain platelet-rich plasma (PRP). In order to remove residual erythrocytes, the PRP samples were again centrifuged at 120 ×g for 7 min. To isolate the platelets and remove the ACD solution, the PRP was centrifuged twice at 350 ×g for 10 min with a washing buffer. The concentration of platelets from the precipitate was adjusted to 3 × 10^8^/mL with Tyrode buffer (137 mM NaCl, 12 mM NaHCO_3_, 5.5 mM glucose, 2 mM KCl, 1 mM MgCl_2_, and 0.3 mM NaHPO_4_, pH 7.4) for the aggregation assay. Platelet preparation was conducted at room temperature. Experimental procedures and protocols involving animals were reviewed and approved by the Ethics Committee of the College of Veterinary Medicine, Kyungpook National University (Daegu, Republic of Korea).

### 2.3. Platelet Aggregation Assay

Platelet aggregation was evaluated as previously described [24]. Aggregation was monitored by measuring light transmission with an aggregometer (Chrono-log, Havertown, PA, USA). The washed platelets (3 × 10^8^/mL) were preincubated at 37°C for 2 min with either CE6 or vehicle and then stimulated with 10 *μ*M ADP. The mixture was further incubated for 5 min with stirring. The vehicle concentration was less than 0.1% to minimize the effect of this reagent.

### 2.4. [Ca^**2+**^]_*i*_ Measurement

The intracellular calcium ion concentration ([Ca^2+^]_*i*_) was measured with Fura-2/AM as previously described [25]. Briefly, the platelets were incubated with 5 *μ*M of Fura-2/AM for 30 min at 37°C and washed. The Fura-2-loaded platelets (3 × 10^8^/mL) were then pre-incubated with CE6 for 2 min at 37°C in the presence of 1 mM CaCl_2_, and subsequently stimulated with ADP for 5 min. Fluorescent signals were recorded using a Hitachi F-2500 fluorescence spectrofluorometer (F-2500, Hitachi, Japan). Light emission was measured at 510 nm, with simultaneous excitation at 340 and 380 nm that changed every 0.5 s. Fura-2 fluorescence in the cytosol measured with the spectrofluorometer was calculated as previously described by Schaeffer and Blaustein [26] with the following formula: [Ca^2+^]_*i*_ 224 nM × (*F* − *F*_min⁡_)/(*F*_max⁡_ − *F*), in which 224 nM is the dissociation constant of the Fura-2-Ca^2+^ complex, and *F*_min⁡_ and *F*_max⁡_ represent the fluorescence intensity levels at very low and very high Ca^2+^ concentrations, respectively. In our experiment, *F*_max⁡_ was the intensity of the Fura-2-Ca^2+^ complex fluorescence at 510 nm after the platelet suspension containing 1 mM of CaCl_2_ had been solubilized with Triton X-100 (0.1%). *F*_min⁡_ was the fluorescence intensity of the Fura-2-Ca^2+^ complex at 510 nm, after the platelet suspension containing 20 mM Tris/3 mM of EGTA had been solubilized with Triton X-100 (0.1%). *F* represented the intensity of Fura-2 complex fluorescence at 510 nm after the platelet suspension which was stimulated with ADP with or without CE6 in the presence of 1 mM CaCl_2_.

### 2.5. ATP Release Assay

Washed platelets (3 × 10^8^/mL) were pre-incubated for 2 min at 37°C with various concentrations of CE6 and then stimulated with 10 *μ*M ADP. After the reaction was terminated, the cells were centrifuged and the supernatants were used for the assay. ATP release was measured in a luminometer (GloMax 20/20, Promega, Madison, USA) using an ATP assay kit (Biomedical Research Service Center, Buffalo, NY, USA) according to manufacturer's instructions.

### 2.6. Serotonin Release Assay

A platelet aggregation assay was carried out as described previously. After terminating the aggregation reaction, the mixture was immediately centrifuged at 12,000 ×g for 5 min at 4°C. The supernatant was collected and serotonin release was measured with a serotonin ELISA kit (Labor Diagnostika Nord GmbH & Co, Nordhorn, Germany) according to the manufacturer's instructions.

### 2.7. Cyclooxygenase-1 (COX-1) Activity Assay

The washed platelets (3 × 10^8^/mL) with 1% protease inhibitor cocktail (Sigma Chemical Co., St. Louis, MO, USA) were sonicated 10 times at sensitivity 100% for 20 s on ice with a model HD2070 sonicator (Bandelin Electronic, Bandelin, Germany) to obtain platelet lysates. The homogenates were centrifuged at 12,000 ×g for 15 min at 4°C to remove cell debris. The supernatant was used to measure COX-1 activity. The platelet lysates were pre-incubated with 330 nM SC-560, a selective COX-1 inhibitor, and with or without various concentrations of CE6 at 37°C for 30 min. COX-1 activity of the treated platelet lysates was then measured with a COX-1 fluorescent assay kit (Cayman Chemical Co., Ann Arbor, MI, USA) according to the manufacturer's protocol.

### 2.8. Measurement of Thromboxane A Synthase (TXAS) Activity

The washed platelets (3 × 10^8^/mL) with 1% protease inhibitor cocktail (Sigma Chemical Co., St. Louis, MO, USA) were sonicated 10 times at sensitivity 100% for 20 s on ice with a model HD2070 sonicator (Bandelin Electronic, Bandelin, Germany) to obtain platelet lysates. Next, the homogenates were centrifuged at 12,000 ×g for 15 min at 4°C to remove cell debris. TXAS activity of the supernatant was then measured. The platelet lysates were pre-incubated with 11 nM ozagrel, a TXAS inhibitor, with or without various concentrations of CE6 at 37°C for 30 min. The reaction was initiated by the addition of prostaglandin H2 (PGH_2_) and allowed to proceed for 1 min at 37°C. The reaction was then terminated by the addition of 1 M citric acid. After neutralization with 1 N NaOH, the concentration of thromboxane B_2_ (TXB_2_), a stable metabolite of TXA_2_, was determined with a TXB_2_ EIA kit (Cayman Chemical Co, Ann Arbor, MI, USA) according to the manufacturer's instructions.

### 2.9. Evaluation of TXB_**2**_ Generation

Washed platelets (3 × 10^8^/mL) were pre-incubated with or without CE6 for 2 min at 37°C in the presence of 1 mM CaCl_2_, and then stimulated with 10 *μ*M ADP for 5 min. The reactions were terminated by adding ice-cold 2.5 mM EDTA and 100 *μ*M indomethacin. After centrifugation at 12,000 ×g for 3 min at 4°C, the supernatant was collected and the concentration of TXB_2_ was measured using a TXB_2_ EIA kit according to the manufacturer's protocol.

### 2.10. Measurement of cAMP Levels

Washed platelets (3 × 10^8^/mL) were incubated at 37°C for 5 min with vehicle (DMSO), CE6 (1, 4, and 8 *μ*M), or forskolin (1 *μ*M) in either the presence or absence of 10 *μ*M ADP with stirring in an aggregometer (Chronolog, Havertown, PA, USA). Immediately after terminating the incubation, the mixture was boiled for 5 min and cooled to 4°C. The samples were then centrifuged at 2,000 ×g for 10 min at 4°C. The concentration of cAMP in the supernatants was determined with a cAMP EIA kit according to the manufacturer's protocol.

### 2.11. Immunoblotting 

Platelet suspensions (3 × 10^8^/mL) were pre-incubated with CE6 or vehicle (0.1% (v/v) DMSO) at 37°C for 2 min. Platelet activation was induced by the addition of 10 *μ*M ADP and the reaction was allowed to proceed for 5 min. After terminating the reaction, lysates were then prepared by solubilizing and centrifuging the platelets in sample buffer (0.125 M Tris-HCl, pH 6.8; 2% SDS, 2% *β*-mercaptoethanol, 20% glycerol, 0.02% bromophenol blue, 1 *μ*g/mL phenylmethylsulfonyl fluoride (PMSF), 2 *μ*g/mL aprotinin, 1 *μ*g/mL leupeptin, and 1 *μ*g/mL pepstatin A). Protein concentration was determined using a BCA assay (Pro-Measure; iNtRON Biotechnology, Seoul, Republic of Korea). Total cell proteins (30 *μ*g) from the platelet lysate were resolved by 10% SDS-PAGE and transferred to nitrocellulose membranes in transfer buffer (25 mM Tris, pH 8.5; 0.2 M glycine; and 20% methanol). The membranes were blocked in TBS-T containing 5% nonfat dry milk and incubated with primary antibody diluted in a blocking solution. The blots were then incubated with horseradish peroxidase-conjugated secondary antibody. Antibody binding was visualized using enhanced chemiluminescence (iNtRON Biotechnology, Seoul, Republic of Korea).

### 2.12. Assessment of Fibrinogen Binding to Integrin *α*_**IIb**_ *β*_**3**_

Fibrinogen Alexa Fluor 488 conjugate binding to washed platelets was quantified by flow cytometry. Briefly, washed platelets (3 × 10^8^/mL) were pre-incubated for 2 min with various concentrations of CE6 at room temperature in the presence of 0.1 mM CaCl_2_. The platelets were then stimulated with ADP for 5 min, immediately incubated thereafter with fibrinogen Alexa Fluor 488 (20 *μ*g/mL) for 5 min, and finally fixed with 0.5% paraformaldehyde at 4°C for 30 min. The platelets were pelleted by centrifugation at 2,000 ×g at 4°C and resuspended in 500 *μ*L PBS. Since the activation of integrin *α*_IIb_ *β*_3_ is largely dependent on the generation of Ca^2+^, nonspecific binding of fibrinogen to integrin *α*_IIb_ *β*_3_ was measured by assessing fibrinogen binding in the presence of the calcium chelator EGTA (1 mM). The fluorescence of each platelet sample was analyzed using a FACS Calibur cytometer (BD Biosciences, San Jose, CA, USA), and data were analyzed using CellQuest software (Becton Dickinson Immunocytometry Systems, San Jose, CA, USA).

### 2.13. Statistical Analysis

Data were analyzed with a one-way analysis of variance followed by a post hoc Dunnett's test in order to measure statistical significance of the differences observed (SAS Institute Inc., Cary, NC, USA). All data are presented as the mean ± standard error of the mean (SEM). *P* values of 0.05 or less were considered to be statistically significant.

## 3. Results

### 3.1. CE6 Inhibits ADP-Induced Platelet Aggregation

**Figure 2:**
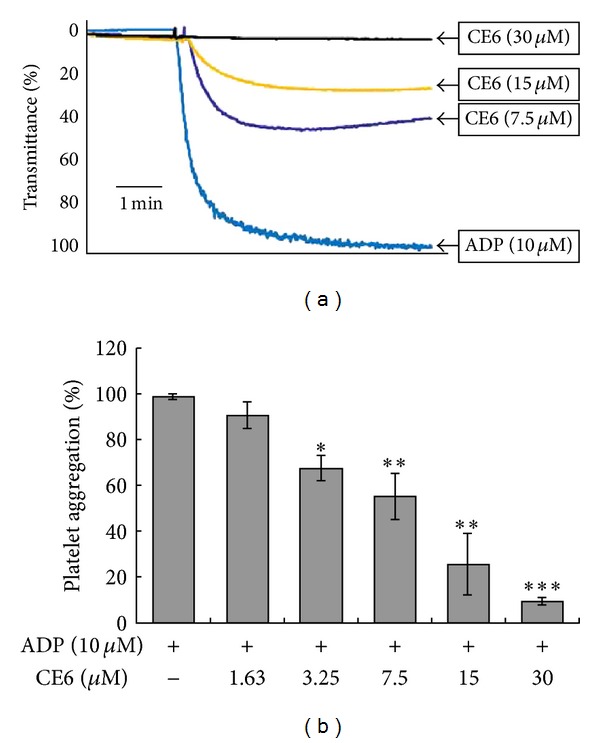
The inhibitory effect of CE6 on platelet aggregation induced by ADP. Platelets (3 × 10^8^/mL) were preincubated with or without CE6 (1.63–30 M) in the presence of 1 mM CaCl_2_ for 2 min at 37°C. The platelet aggregation was then induced by 10 *μ*M ADP and measured with a turbidimetric aggregometer. The aggregation reaction was terminated after 5 min, and the percent aggregation rate was calculated. Each graph shows the mean ± SEM of four independent experiments. *P < 0.05, **P < 0.01, and ***P < 0.001 compared to the agonist control.

We first determined whether CE6 affected platelet aggregation induced by 10 *μ*M ADP. CE6 significantly inhibited ADP-induced platelet aggregation in a concentration-dependent manner with an IC_50_ of 12.53 ± 2.79 *μ*M (Figure 2).

### 3.2. CE6 Prevents ADP-Induced [Ca^**2+**^]_*i*_ Elevation

**Figure 3:**
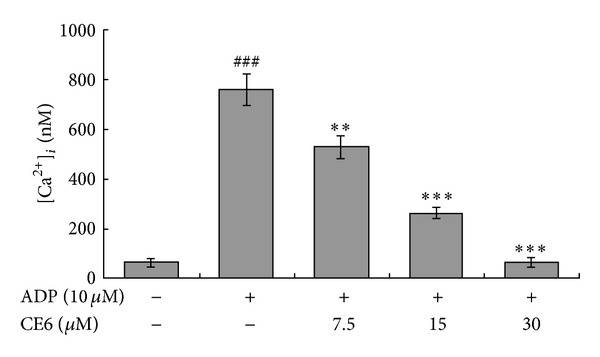
The inhibitory effect of CE6 on [Ca^2+^]_i_ increased by ADP. Washed platelets (3 × 10^8^/mL) were incubated with calcium fluorophore (5 M, Fura-2/AM) and stimulated with 10 *μ*M ADP. [Ca^2+^]_i_ was then measured as described in Section 2. The results are presented as the mean ± SEM of three independent experiments. **P < 0.01 and ***P < 0.005 compared to the agonist control. ^###^ *P* < 0.001 compared to the basal level.

Since the mobilization of calcium is a crucial step for platelet activation and degranulation [27], we examined the effect of CE6 on the elevation of [Ca^2+^]_*i*_ induced by ADP. As shown in Figure 3, ADP (10 *μ*M) increased [Ca^2+^]_*i*_ to 750 nM. This was markedly suppressed by CE6 in a concentration-dependent manner. Our results suggest that the inhibition of platelet aggregation by CE6 is potentially mediated by the regulation of [Ca^2+^]_*i*_.

### 3.3. CE6 Inhibits ATP Release from ADP-Activated Platelets

**Figure 4:**
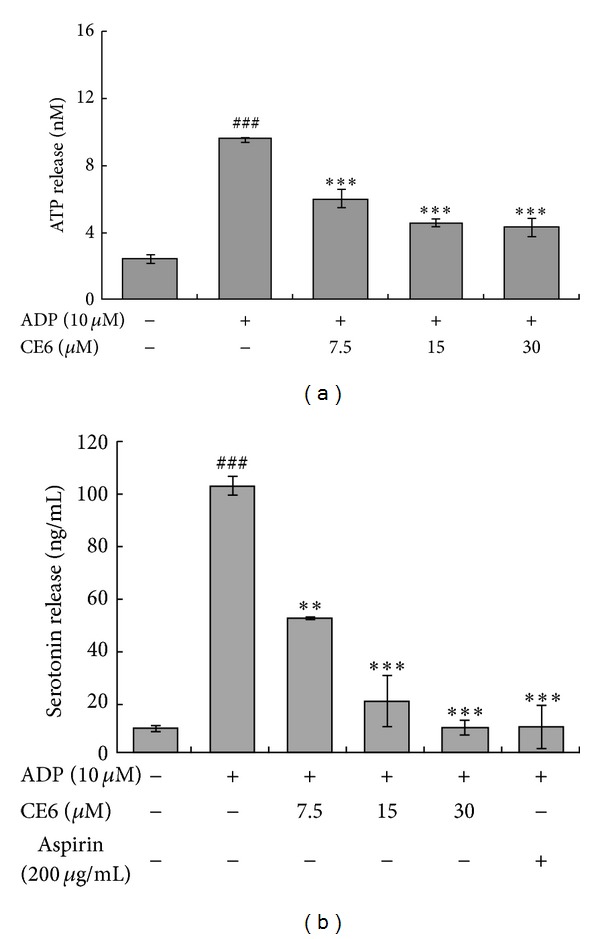
Effects of CE6 on granule secretion from the ADP-activated platelets. Washed platelets (3 × 10^8^/mL) were pre-incubated with CE6 at the indicated concentrations and stirred in an aggregometer for 2 min prior to stimulation with ADP for 5 min. The reaction was terminated, and an ATP release assay (a) and serotonin release assay (b) were performed. Bar graphs show the mean ± SEM of at least four independent experiments. **P < 0.01 and ***P < 0.001 compared to the agonist control. ^###^ *P* < 0.001 compared to the basal level.

Since the contents of dense granules are rapidly released as an early event of platelet activation [28], we measured ATP secretion induced by ADP as an index of dense granule secretion. Compared to treatment with vehicle, CE6 significantly inhibited ATP release from ADP-stimulated platelets (Figure 4).

### 3.4. CE6 Reduces Serotonin Release from ADP-Activated Platelets

**Figure 5:**
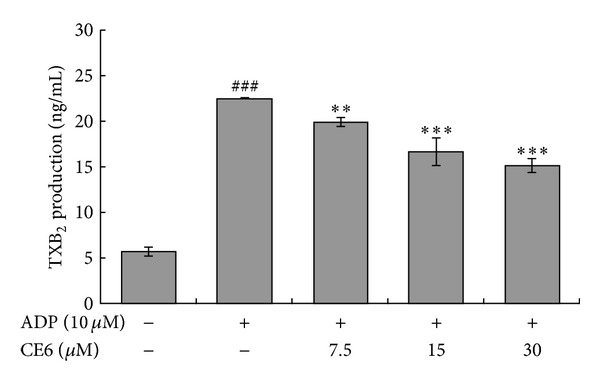
Effect of CE6 on ADP-induced TXB_2_ generation. Platelets (3 × 10^8^/mL) were pre-incubated with or without CE6 (7.5–30 M) in the presence of 1 mM CaCl_2_ for 2 min at 37°C. The platelets were then stimulated with 10 *μ*M ADP for 5 min at 37°C. After terminating the aggregation reaction, the supernatant was collected and TXB_2_ production was measured using a TXB_2_ EIA kit according to the manufacturer's instructions. Bar graphs show the mean ± SEM of three independent experiments performed. **P < 0.01 and ***P < 0.005 compared to the agonist control. ^###^ *P* < 0.001 compared to the basal level.

Serotonin (5-hydroxytryptamine) accumulates in platelet dense granules and is released during the initial stage of platelet activation with various ligands such as collagen, thrombin, and ADP [29, 30]. As shown in Figure 5, CE6 significantly suppressed ADP-induced serotonin release from the rat platelets in a concentration-dependent manner. Taken together, our findings showed that CE6 markedly inhibited degranulation during the early step of ADP-induced platelet activation.

### 3.5. CE6 Suppresses TXA_**2**_ Production

**Figure 6:**
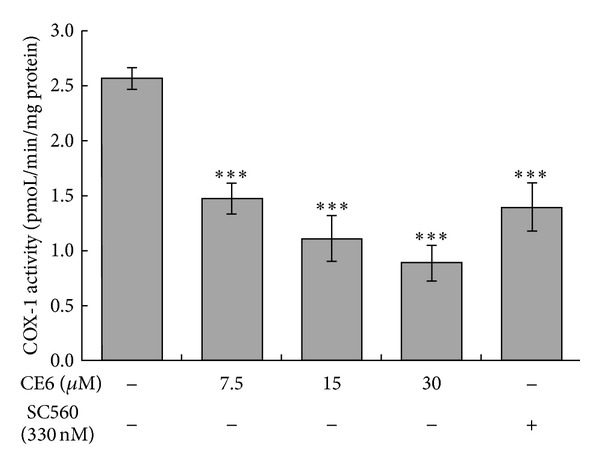
Effect of CE6 on COX-1 activity in the intact platelets. Platelets (3 × 10^8^/mL) with protease inhibitor cocktail were sonicated 10 times at sensitivity 100% for 20 s on ice with sonicator. The homogenates were centrifuged at 12,000 × g for 15 min at 4°C to remove cell debris. The supernatant was used to measure COX-1 activity. The platelet lysates were pre-incubated with or without various concentrations of CE6 at 37°C for 30 min. COX-1 activity of the treated platelet lysates was then measured with a COX-1 assay kit according to the manufacturer's protocol. Bar graphs show the mean ± SEM of three independent experiments. ***P < 0.005 compared to the agonist control.

TXA_2_, a lipid mediator, is a key element that amplifies activation signals. To examine the effects of CE6 on TXA_2_ production, we quantified the levels of TXB_2_, a stable metabolite of TXA_2_ in CE6-treated platelets. As presented in Figure 6, ADP greatly increased TXB_2_ generation five times more compared to the basal level. Production of this metabolite was gradually reduced by CE6.

### 3.6. CE6 Inhibits COX-1 Activity but Not TXAS Activity

TXA_2_, a powerful aggregating agent that acts as an autacoid, can be produced via sequential activation of COX-1 and TXAS. First, COX-1 catalyzes the conversion of arachidonic acid into PGH_2_, which is transformed into TXA_2_ with the aid of TXAS [23]. We determined whether CE6 modulated the activities of COX-1 and TXAS in unstimulated rat platelets. Surprisingly, CE6 inhibited COX-1 activity in a concentration-dependent manner compared to SC-560, a selective COX-1 inhibitor (Figure 6). However, CE6 did not affect TXAS activity (data not shown).

### 3.7. CE6 Increases cAMP Production in Resting and ADP-Activated Platelets

**Figure 7:**
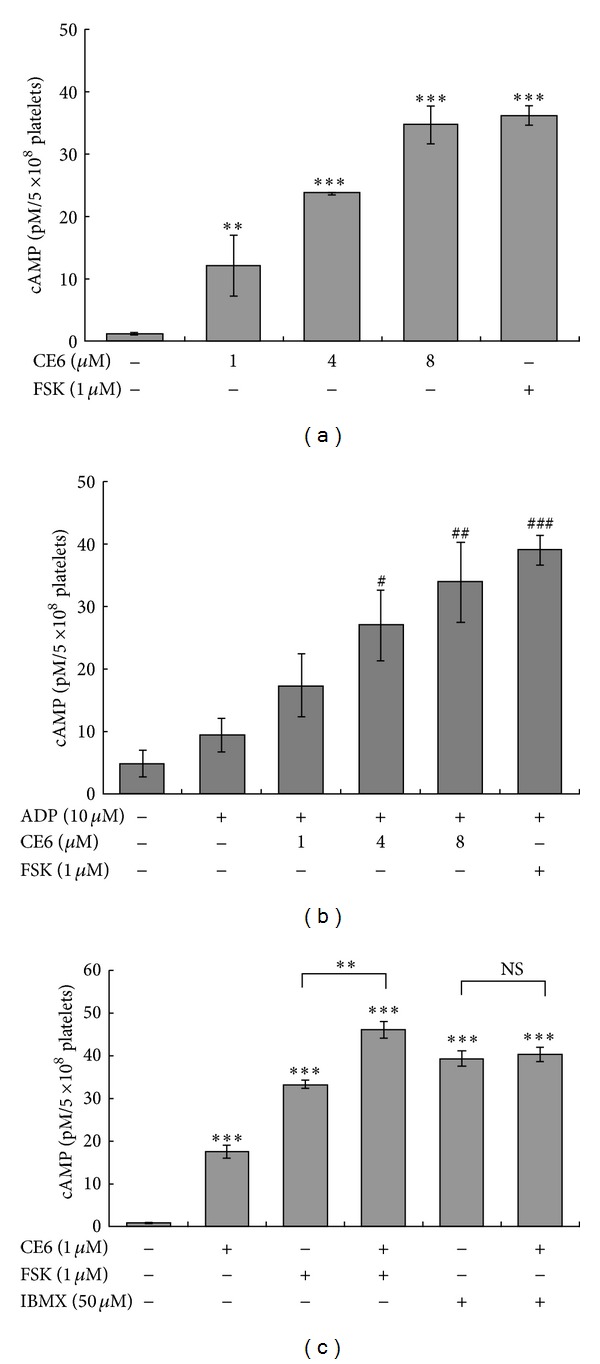
Effects of CE6 on intracellular cAMP concentrations. Washed platelets (3 × 10^8^/mL) were stirred with vehicle (DMSO) or CE6 (at the indicated concentrations), forskolin, or IBMX in an aggregometer and stimulated with 10 *μ*M ADP for 5 min. cAMP levels were then measured with an immunoassay as described in Section 2. CE6 significantly increased cAMP accumulation in a concentration-dependent manner (a). Co-incubation with CE6 and forskolin but not IBMX synergistically increased cAMP levels (b). The results are presented as the mean ± SEM of at least three independent experiments. **P < 0.01, and ***P < 0.005 compared to the negative control. ^#^ *P* < 0.05, ^##^ *P* < 0.01, and ^###^ *P* < 0.001 compared to the ADP-activated control. NS: not significant.

As shown in Figure 7(a), preincubation with CE6 significantly increased the cAMP levels in intact platelets. At a concentration of 8 *μ*M, CE6 increased cAMP concentrations to 95% of that observed in cells treated with forskolin. However, ADP has a little effect on cAMP production while CE6 augmented cAMP production in ADP-activated platelets with the similar extent in resting platelets (Figure 7(b)). Furthermore, coincubation with CE6 and 1 *μ*M forskolin, a direct AC activator, promoted the increase of cAMP levels induced by CE6 (Figure 7(c)). However, co-incubation with CE6, 3-isobutyl-1-methylxanthine (IBMX), a broad-spectrum PDE inhibitor, had a little synergistic effect on cAMP production.

### 3.8. CE6 Inhibits the Phosphorylation of MAPKs, PI3K, and Akt and Increases Expression of the PKA Catalytic Subunit

**Figure 8:**
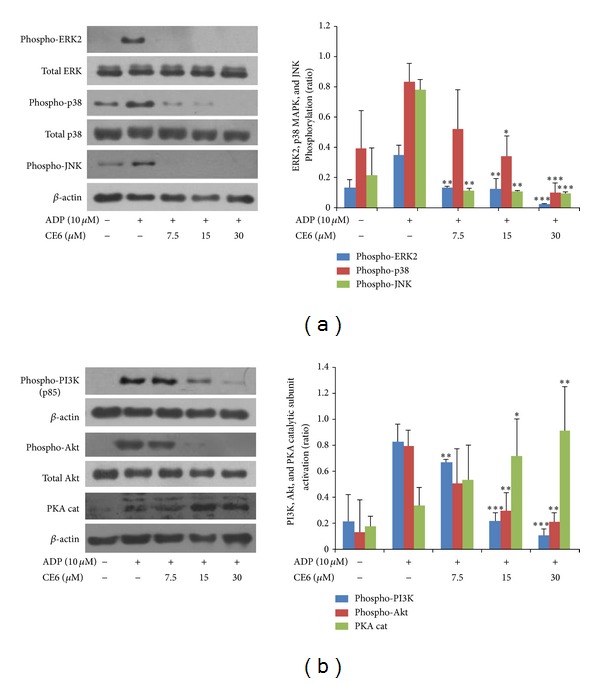
Effects of CE6 on ADP-induced phosphorylation of MAPKs, PI3K, and Akt and on expression of the PKA catalytic subunit. Washed platelets (3 × 10^8^/mL) were pre-incubated for 2 min with vehicle or CE6 at the indicated concentration. The platelets were then stimulated with 10 *μ*M ADP for 5 min at 37°C. After terminating the reactions, total cell proteins were extracted. The proteins were separated by SDS-PAGE and transferred onto *nitrocellulose membranes*. The membranes were then probed with antibodies against phospho-p44/42, p44/42, phospho-p38, p38, phospho-SAPK/JNK, and *β*-actin (a); and phospho-PI3K, phospho-Akt, Akt, PKA*α*/*β*/*γ* catalytic subunit, and *β*-actin (b). Antibody binding was visualized by chemiluminescence. All immunoblots are representative of three or four independent experiments.

In order to elucidate the mechanism underlying the effects of CE6 on platelets, we further examined the phosphorylation of downstream signaling molecules including MAPKs, PI3K, and PKA. MAPKs include extracellular signal-related kinase 1/2 (ERK1/2 or p44/42), p38 MAPK, and JNK, which are all expressed in rat platelets. Our immunoblot analysis revealed that phosphorylation of all three MAPKs was almost completely blocked by pre-incubation with CE6 (Figure 8(a)).

It is well-established that PI3K plays crucial role in dense granule secretion along with signaling associated with secondary mediators such as ADP and TXA_2_ [7]. In addition, PI3K and Akt are known to be phosphorylated by G_i_-coupled receptor activation [4]. We therefore determined whether CE6 affected PI3K and Akt phosphorylation. Pre-incubation with CE6 significantly inhibited the phosphorylation of PI3K and Akt in rat platelets (Figure 8(b)).

**Figure 9:**
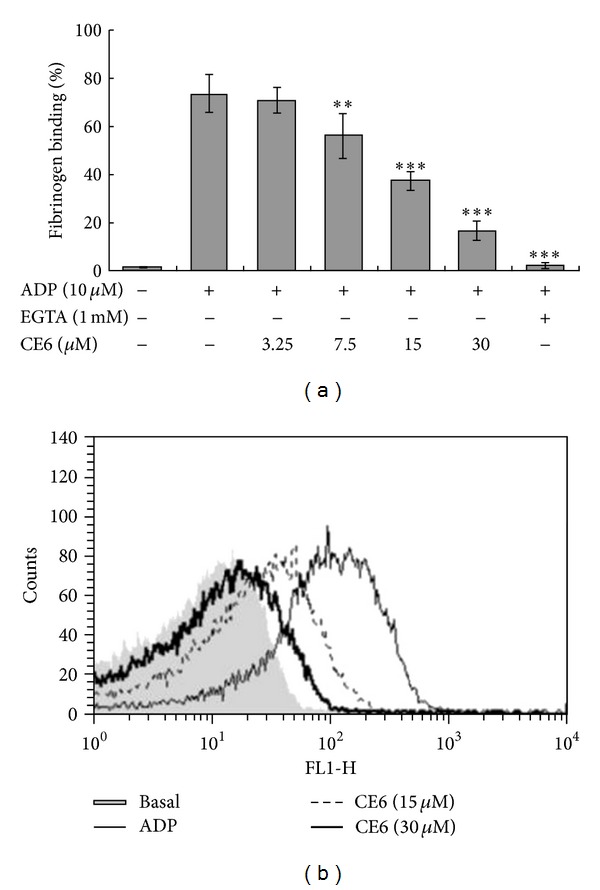
Effects of CE6 on fibrinogen binding to integrin *α*_IIb_ *β*_3_ in ADP-activated platelets. The inhibitory effects of CE6 on fibrinogen binding to integrin *α*_IIb_ *β*_3_ in ADP-stimulated platelets were measured by flow cytometry (a). Washed platelets (3 × 10^8^/mL) were pretreated with vehicle (DMSO) or CE6 at concentrations ranging from 3.25 *μ*M to 30 *μ*M. ADP (10 *μ*M) was then incubated with human fibrinogen labeled with Alexa Fluor 488 (20 *μ*g/mL) for 5 min. The cells were subsequently fixed with 0.5% paraformaldehyde at 4°C for 30 min. Graphs showing fluorescent intensity present the data from one experiment but are representative of four independent trials. Data are expressed as the mean fluorescence intensity (MFI) of fibrinogen-positive platelets. Each graph presents the results expressed as percent of gated (A). **P < 0.01 and ***P < 0.005 compared to the agonist control.

Since pre-incubation with CE6 increased cAMP production in unstimulated platelets, we next explored whether PKA is activated by pretreatment with CE6. As shown in Figure 9(b), CE6 significantly enhanced expression of the PKA catalytic subunit.

### 3.9. CE6 Attenuates Fibrinogen Binding to Integrin *α*_IIb_ *β*_**3**_

One consequence of ADP-induced platelet activation is a conformational change of integrin *α*_IIb_ *β*_3_ [31] that binds to serum fibrinogen. Concomitant stimulation of two ADP receptors, P2Y_1_ and P2Y_12_, causes integrin *α*_IIb_ *β*_3_ to assume an active conformation [32]. To determine whether CE6 affects the activation of *α*_IIb_ *β*_3_, washed platelets were pre-incubated with different concentrations (3.25~30 *μ*M) of CE6 and incubated with fibrinogen bound to Alexa Fluor 488. Specific binding of the labeled fibrinogen was then measured. We found that CE6 inhibited fibrinogen binding in a concentration-dependent manner (Figures 9(a) and 9(b)).

## 4. Discussion

**Figure 10:**
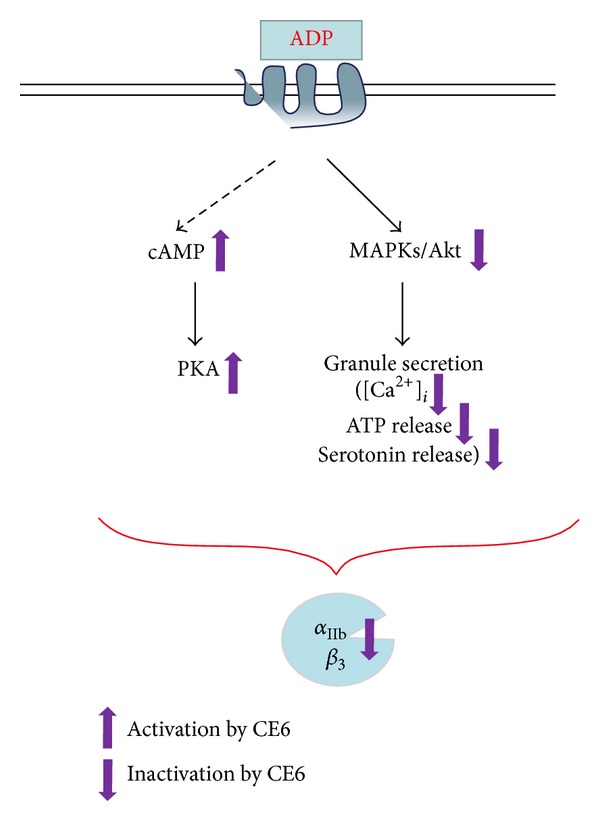
Schematic of CE6 antiplatelet activity and the underlying mechanism of action.

In the present study, we evaluated the inhibitory effects of CE6 on platelet aggregation and elucidated downstream pathways involved in the inhibition of ADP-induced platelet activation by CE6. ATP and serotonin secretion was inhibited by CE6. This compound also regulated [Ca^2+^]_*i*_. Moreover, the phosphorylation of the three MAPKs, PI3K, and Akt was inhibited, and fibrinogen binding to integrin *α*_IIb_ *β*_3_ was attenuated (Figure 10).

We demonstrated that CE6 prevented ADP-induced [Ca^2+^]_*i*_ elevation in a concentration-dependent manner and significantly suppressed dense granule secretion. Upon ADP stimulation, a rapid rise in [Ca^2+^]_*i*_ occurs due to PI3K activation [2]. Guidetti et al. also demonstrated that the inhibition of P2Y_12_ (a G_i_-coupled ADP receptor) completely prevents PKC activation and suppresses pleckstrin phosphorylation [2]. These findings suggest that CE6 inhibits calcium mobilization at least partially via the inhibition of DAG-PKC activity. Increased [Ca^2+^]_*i*_ subsequently triggers platelet granule secretion (i.e., and dense granules) [33]. ATP released from dense granules causes a rapid influx of calcium by sensitizing the ionotropic receptor P2X1 [34]. On the other hand, Ca^2+^ chelation abolishes ATP release, indicating functional complementarities [35]. In addition, released granule contents (i.e., ATP and serotonin) help amplify platelet activation [29, 33, 36]. Taken together, our results along with data from the literature suggest that CE6 exerts broad inhibitory effects on granule secretion such as calcium, ATP, and serotonin, which mediated the antiplatelet activities of CE6.

TXA_2_ is an unstable aggregating factor, of which production was significantly inhibited by pre-incubation with CE6 in ADP-activated platelets. TXA_2_ is an important mediator generated by the initial activation of phospholipase A_2_. Recent studies have shown that activation of the P2Y_1_ purinergic receptor (G_q_-coupled ADP receptor) enhances TXA_2_ receptor (TP receptor) activation [37], and the TP receptor subsequently promotes ADP secretion [38]. These findings suggest that cross-activity exists between G protein-mediated receptors. Given that the network between ADP and TXA_2_ enables the rapid formation of a haemostatic plug, the inhibition of TXA_2_ generation seems to play an important role in mediating the inhibitory effect of CE6 on ADP-induced activation. TXA_2_ is produced by sequential activation of COX-1 and TXAS from arachidonic acid, a byproduct of phospholipid breakdown. As presented in Figure 6, CE6 markedly suppressed COX-1 activity but not that of TXAS (data not shown). This result suggests that the inhibition of TXA_2_ production (determined by measuring the TXB_2_ metabolite levels) by CE6 is likely due to decreased availability of PGH_2_, a substrate of TXAS.

 Mammalian platelets express three MAPKs: ERK1/2, p38-MAPK, and JNK. Previous studies showed that ERK has a crucial role in TXA_2_ generation [11, 19]. Moreover, ERK activation following GPIb stimulation leads to integrin *α*_IIb_ *β*_3_ activation [19]. p38-MAPK and ERK are known to have important roles in granule secretion and TXA_2_ release, suggesting that the effects of these two factors are secondary to the effect on granule secretion [39]. Our results indicated that CE6 suppresses dense granule secretion, TXA_2_ formation, and *α*_IIb_ *β*_3_ activation (at least in part) through inhibition of the ERK and p38-MAPK pathways. On the other hand, previous studies have presented and demonstrated that JNK−/− platelets are associated with increased bleeding time, decreased integrin *α*_IIb_ *β*_3_ activation, and severe granule secretion impairment [40]. Therefore, it seems that the inhibition of JNK phosphorylation plays an important role in the platelet activation process.

 The most noteworthy finding from the present study was that cAMP production was elevated by CE6 treatment in resting or ADP-activated platelets. P2Y_12_ receptor is capable of inhibiting AC, thereby promoting platelet aggregation [2]. Additionally, ADP suppresses intracellular cAMP levels increased by constitutive activation of AC in intact platelets [8]. Srinivasan et al. also reported that adenosine-based P2Y_12_ (coupled to G_s_) antagonists inhibit platelet aggregation, but adenosine-based P2Y_1_ (coupled to G_q_) antagonists do not [8]. As such, considerable evidence has accumulated supporting the theory that cAMP has a broad inhibitory effect on platelet activation [24, 41]. With this knowledge, we tested our hypothesis that CE6 would raise cAMP levels in the rat platelets. As expected, CE6 markedly elevated cAMP concentrations compared to forskolin, suggesting that the inhibitory effect of CE6 on platelet activation is mediated by cAMP. Moreover, the synergistic effect we observed with the AC activator but not the PDE inhibitor indicates that the elevation of cAMP production by CE6 is not due to the inhibition of cAMP degradation through PDE activity suppression.

It is well known that the target of cAMP is PKA, which is composed of a regulatory subunit dimer and two catalytic subunits [1, 6]. Indeed, treatment with CE6 at concentrations that inhibited platelet aggregation increased the expression of the active PKA catalytic subunit in the present study. Our finding indicated that activation of the cAMP-PKA pathway is responsible for the inhibition of platelet aggregation by CE6. Unexpectedly, fibrinogen binding to integrin *α*_IIb_ *β*_3_ was much more affected by CE6 treatment compared to intracellular cAMP levels. Several previous studies have reported that inhibition of AC or ADP receptors does not induce platelet aggregation [32, 42]. Thus, the cAMP pathway elicited by CE6 might be of potentiating effect rather than direct effect on integrin signaling.

 The inhibition of integrin activation is of great interest for the development of antiplatelet drugs. As a consequence of active integrin conformation, outside-in signaling is a major driving force for complete platelet aggregation [43]. Reversible cell aggregation is eventually undergoing stabilization, followed by outside-in signaling of fibrinogen. Several lines of research have demonstrated that platelet aggregates are completely or partially disassembled in ADP- or collagen-stimulated platelets through either blocking of the P2Y_12_ receptor or suppression of PI3K [8, 44]. Based on these findings and our data, we propose that ADP-activated PI3K plays an important role in integrin activation, likely via G_*i*_-coupled signaling.

Alternatively, it was previously shown that PKC activation is inhibited by either blocking the P2Y_12_ receptor or inhibiting PI3K activity [45]. These findings suggest that PKC holds a pivotal role in the PI3K-Akt signaling pathway. As discussed previously, the inhibition of PKC activity likely influenced the negative effects of CE6 on platelet activation. During the late stage of integrin activation, the ADP-P2Y_12_ receptor helps regulate thrombus stabilization through the activation of PI3K and Akt, which inhibits cAMP production [44]. Kim et al. also reported that cAMP downregulates the activity of Akt in COS cell by interfering PtdIns-3,4,5-P_3_ formation [46], demonstrating the essential role of ADP in activation of the Akt pathway [4]. Results from our study suggest that the suppression of fibrinogen binding to *α*_IIb_ *β*_3_ by CE6 is mediated by decreased activation of PI3K and Akt.

## 5. Conclusion

As summarized in Figure 10, we demonstrated that CE6 inhibited ADP-induced platelet aggregation, increases of [Ca^2+^]_*i*_, TXA_2_ production, and dense granule secretion (i.e., ATP and serotonin release) in a concentration-dependent manner. The phosphorylation of the three MAPKs (ERK1/2, p38-MAPK, and JNK) was also markedly inhibited by CE6 treatment. While the PI3K-Akt pathway was suppressed by CE6, the cAMP-PKA pathway was activated. Based on our data, we propose that CE6 acts through the cAMP-PKA pathway to attenuate the expression and activity of intracellular signaling molecules and suppress the activation of integrin *α*_IIb_ *β*_3_ activated by PI3K-Akt signaling, thereby preventing the aggregation of ADP-stimulated platelets. The development of innovative antiplatelet drugs has been focused on the inhibition of ADP and TXA_2_ signaling [37]. Thus, CE6 could be developed as a therapeutic agent for preventing thrombosis and ischemia.

## Abbreviations

CE6: Chlorin e6
MAPK: Mitogen-activated protein kinase
TXA_2_: Thromboxane A_2_
PI3K: Phosphatidylinositol 3-kinase
GPCR: G-protein coupled receptor
DAG: Diacylglycerol
PKA: Protein kinase A
PKC: Protein kinase C.
